# Changes in neurobiological markers of reactivity to alcohol-related stimuli in alcohol-dependent patients after two years of treatment

**DOI:** 10.3389/fpsyt.2025.1613169

**Published:** 2025-06-18

**Authors:** Gabriel Rubio, Marta Marín, Cristina Martín-Arriscado Arroba, Enrique Rubio-Escobar, Francisco Arias, Abraham B. Torregrosa, Jorge Manzanares

**Affiliations:** ^1^ Instituto de Investigación Sanitaria Hospital 12 de Octubre (imas12), Madrid, Spain; ^2^ Departamento de Psiquiatría, Facultad de Medicina, Universidad Complutense de Madrid, Madrid, Spain; ^3^ Servicio de Psiquiatría, Hospital Universitario 12 de Octubre, Madrid, Spain; ^4^ Red de Investigación en Atención Primaria de Adicciones, Instituto de Salud Carlos III, Ministerio de Ciencia, Innovación y Universidades (MICINN) and Fondo Europeo de Desarrollo Regional (FEDER), Madrid, Spain; ^5^ Instituto de Neurociencias, Universidad Miguel Hernández-Consejo Superior de Investigaciones Científicas (CSIC), Alicante, Spain; ^6^ Instituto de Investigación Sanitaria y Biomédica de Alicante (ISABIAL), Alicante, Spain

**Keywords:** alcohol, recovery, startle, cortisol, stress

## Abstract

**Background:**

Salience and cortisol response following exposure to alcohol-related stimuli (ARS) are considered markers of vulnerability and status in alcohol use disorders (AUD). Negative emotionality may also maintain alcohol consumption by negative reinforcement. What happens to these parameters once sustained symptom remission has been achieved is unknown. This study investigated changes in these variables after 2 years of outpatient treatment and compared them with a healthy control group.

**Methods:**

Patients with AUD (n=154) were assessed at baseline (T0) and after 2 years of treatment (T2). Salience was determined by the magnitude of the startle reflex when subjects were exposed to pictures of alcoholic beverages and scenes related to alcohol consumption. Salivary cortisol was measured before and after this exposure. Negative emotionality was assessed using anxiety, depression, and impulsivity scales. A group of 138 healthy controls was selected based on sample characteristics. Changes in patient (case) population variables at T0 and T2 were assessed using the Student’s t-test for related samples. A repeated measures variance model (RMMM) was also fitted to the logarithmic values ​​over time.

**Results:**

Startle reflex response and salivary cortisol levels before and after ARS viewing did not change significantly in men or women between T0 and T2. Post-exposure salivary cortisol changes were lower in the male group and in those who consumed alcohol during the treatment period. Scores on negative emotionality scales decreased significantly from T0 to T2. At the end of the study (T2), patients still had altered salience values, cortisol reactivity and negative emotionality compared to the control group.

**Conclusions:**

The absence of changes in these neurobiological markers confirms the chronicity of moderate-severe AUD and the risk of relapse despite the end of treatment. These findings question the duration of alcohol dependence approaches and suggest the need to look for longer-term intervention alternatives.

## 1 Introduction

Alcohol use disorder (AUD) is considered a chronic disease with frequent relapses throughout the patient’s life, with significant medical, family and social repercussions (1). Addiction has been conceptualized as a three-stage cycle – binge/intoxication, withdrawal/negative affect, and preoccupation/anticipation – that worsens over time and involves allostatic changes in the brain’s reward and stress systems (2). In these three stages, the brain reward circuit (stage 1: positive reinforcement), the amygdala and the HPA axis (Stage 2: alcohol use by negative reinforcement) and the prefrontal cortex (Stage 3: anticipation of alcohol use in specific external or internal contexts) are mainly involved (1, 2).

When evaluating substance use disorders (SUDs), different biological and clinical markers related to these reward and stress systems have been proposed (3, 4). Regarding AUD, increased appetitive responses and attentional bias towards alcohol-related cues, as well as increased stress response reactivity following exposure to these cues, have been suggested (4). In addition, negative emotional states (anxiety, impulsivity and depression) associated with the addictive process will contribute to the maintenance of alcohol consumption through negative reinforcement (2). Indeed, there is agreement in considering that modifications in these domains determine the greater or lesser risk for the development of addiction and the prognosis of short-term treatments (4).

Although AUD has been considered a chronic relapse-prone disorder [APA 2013 (5)], no studies have explored long-term modifications in these markers. For this reason, we do not know whether the achievement of abstinence leads to changes in the magnitude of these markers or whether, on the contrary, despite the patient being asymptomatic for a long time, they remain unchanged. Answering this question could help to evaluate the efficacy of therapeutic interventions, focusing on each of these dysfunctional markers and improving the prognosis of patients with AUD.

Given that within these domains, different assessment tools can be used, another challenge for researchers in the field of alcohol addiction lies in choosing the most appropriate assessment technique for each domain (3). In our experience, the characterization of cognitive, motivational and behavioral responses to alcohol-related stimuli (ARS) is the keystone for improving the prognosis of pharmacological and psychotherapeutic treatments used in alcohol-dependent patients. Different methods have been used in the field of AUD to objectively assess the subject’s appetitive responses to alcohol-related stimuli (ARS) (3). Some techniques are based on the attentional bias towards these cues (6); others determine the difficulty in initiating approach behaviors towards ARS (7), and others evaluate the reactivity of specific brain areas – RMf techniques – after visualization of alcohol-associated stimuli (8). We have chosen the startle reflex modulation paradigm because it has been extensively tested in animal and human studies. It is inexpensive and straightforward (9), and our group has extensive experience (10–12).

The *startle reflex* consists of a diffuse skeletal-muscular response that occurs, similarly in many species, following intense and unexpected stimuli (9). One of these paradigm’s most used motor components is the eyeblink, as it appears within 30–50 milliseconds of the eliciting stimulus and is rarely subject to voluntary control. The magnitude of the eyeblink response can be modulated by the presentation of other stimuli, such that the display of stimuli with aversive valence increases the magnitude of the startle response. In contrast, appetitive stimuli decrease it (9).

Studies using this paradigm in patients with AUD have indicated the magnitude of the startle reflex while visualizing ARS was lower than that shown after viewing pleasant, neutral or aversive stimuli, indicating the ability of ARS to facilitate a motivational approach response to those stimuli (10, 13–15). This kind of response has also been found in seeking treatment individuals who had been abstinent for several weeks (10, 11) and was also a predictor of poor prognosis of detoxification treatment (10, 11, 16, 17). However, we do not entirely know whether this reactivity while visualizing ARS, which could explain the recovery of patients after treatment, modifies or not after prolonged periods of abstinence (e.g., after sustained remission-more than one year asymptomatic).

Different studies have investigated the modulation of the *cortisol response* following exposure to alcohol-related contexts and found significant plasma cortisol elevations compared to neutral contexts (18–21). ARS also elicited increased cravings and a blunted cortisol response, leading to greater behavioral motivation toward alcohol (19, 21). However, except for Munro’s study, which only included 18 alcohol-dependent males (22), human studies have not assessed whether these hypothalamic-pituitary-adrenal (HHA) axis dysfunctions are maintained or normalized beyond 6–8 weeks post-detoxification (23, 24). For this reason, it is unknown whether the HHA axis response following an individual’s exposure to alcohol- or stress-associated stimuli remains altered or recovers after a prolonged period of abstinence. In this study, we have chosen the determination of cortisol in saliva because it correlates well with the activation of the hypothalamic-pituitary-adrenal axis (25) and because it is a minimally invasive technique (18, 19).

The importance of *negative emotionality* in these patients stems from the high comorbidity of alcohol addiction with anxiety, depression and impulsivity disorders (26–30). Several studies have found that compared to control populations, alcohol-dependent patients exhibit elevated anxiety, depression and impulsivity scores (30, 31), which decrease during the first months and years of treatment (32, 33). However, the persistence of some of the variables included in the concept of negative emotionality could be explained by the presence of a psychiatric comorbidity before the development of addiction (30, 31) and, therefore, would represent a constant vulnerability marker in many of the people who seek treatment for their alcohol dependence.

In conclusion, all these results reveal a significant lack of knowledge of how these neurobiological processes mentioned above modify during long-term recovery. It can be hypothesized that after years of abstinence, these processes could have normalized significantly so that they do not pose a challenge to maintaining a sober lifestyle or that, if these processes remain unmodified, patients would have been able to counteract the role of these altered processes with other strategies that help them to support recovery.

The purpose of this study was to explore the progress of startle response reactivity during the visualization of ARS, stress reactivity by determining salivary cortisol before and after exposure to alcohol-associated images (34), and anxiety, depression and impulsivity scores, as well as valence, activation and dominance motivated by the visualization of alcohol films in alcohol-dependent patients from a baseline assessment after 6–12 weeks of detoxification to a follow-up assessment two years later after completing an abstinence-oriented therapeutic program.

According to previous research that has focused on the acute phase of alcohol dependence treatment (studies have extended between 3–12 months), it was hypothesized that: a) cue-reactivity to alcohol-related cues assessed by the startle reflex modulation paradigm would not be significantly modified during the study period (15); b) cortisol levels before and after exposure to alcohol-related visual stimuli would be attenuated after two years of treatment (22); c) scores in negative emotionality and subjective assessment of alcohol films would improve after two years in treatment (30, 31); d) after two years of treatment, the variables studied in patients would still be altered compared to a control group, for negative emotionality variables see references (30, 31).

## 2 Methods

### 2.1 Patients

**Figure 1:**
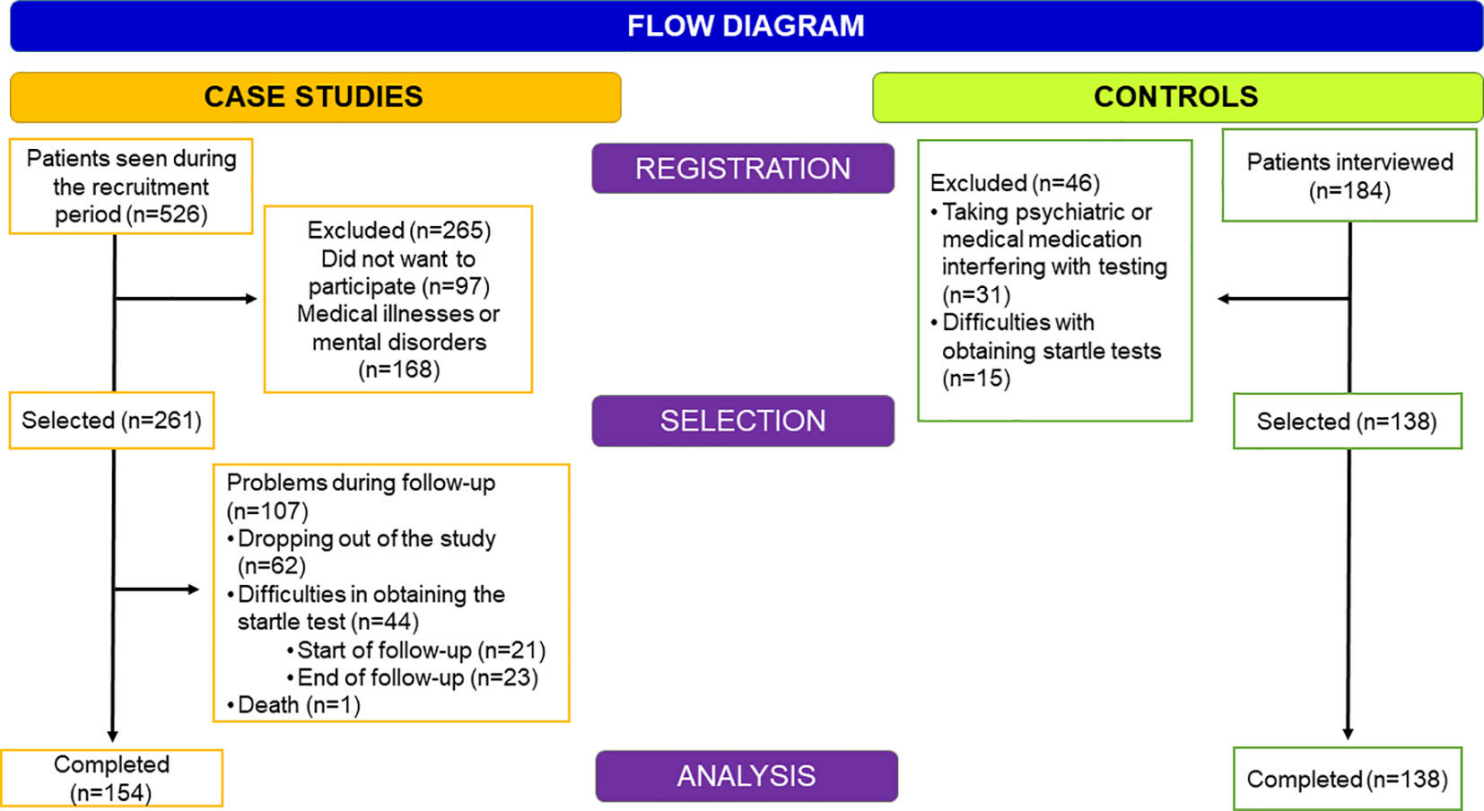
Timeline of evaluations throughout the treatment period.

The sample was selected from patients attending the Alcoholism Treatment Program at Hospital 12 de Octubre between 2019 and 2021 (see **Figure 1**). Of a total of 526 patients who attended the program, 256 were excluded or declined to participate for the following reasons: 1) Refusal to participate (n=97), 2) Concomitant medical illnesses that may hinder the results of the stress test (n=65, 6 of which had a cognitive impairment assessed by TEDCA (35), and 3) Mental illnesses in which it was advisable to maintain psychiatric medication and therefore could interfere with the startle tests (n=103). Of those selected patients (n=261) during the follow-up, 107 could not be included in the final sample for the following reasons: 1) Errors in the acquisition of the startle tests (in the baseline evaluation, n=21 and in the final assessment, n=23), 2) Dropped out of the treatment program (n=62) and in one case due to death (n=1). Hence, the final sample used for this study consisted of 154 subjects.

To facilitate comparisons between patients and healthy controls, the latter was matched according to several variables that could affect results regarding cortisol levels (age, sex, body mass index [BMI], and cigarette consumption). Subjects with AUDIT scores higher than 8 in males or 5 in females were excluded (36). In addition, control subjects taking psychotropic medication or having medical conditions that would interfere with stress testing (n=31) were excluded. Fifteen subjects had to be excluded because their startle records were inadequate for analysis. The final group of controls consisted of 138 subjects.

To determine the appropriate sample size, we considered our group’s previous studies using the startle reflex modulation paradigm (10, 11) and psychological assessments of anxiety and depression (30, 31). Retrospective power analyses were performed based on paired mean comparisons with a 95% confidence level and a sample size of 154 pairs. The sample size of 154 pairs is adequate to detect differences in most variables of interest (GGT, startle, depression and cortisol reactivity) (Range of power calculations 70–99,5%).

### 2.2 Experimental design

This follow-up study included subjects who underwent therapeutic outpatient treatment at our center for 2 years years until discharge (30). This treatment program consisted of a multimodal approach integrating four sections developed over 24 months. Firstly, detoxification and motivation towards an abstinence program (2–3 months) are applied. After finishing detoxification, patients were offered a pharmacological treatment to prevent relapse for at least 9 months (naltrexone or disulfiram or none of them). During the first 2–3 months post-detoxification, they were included in a therapeutic group based on the motivational model and oriented towards abstinence (37). Once detoxification was completed, the patients were given information about the study, and they signed the informed consent form. Secondly, a relapse prevention program was provided based on a cognitive behavioral model by Marlatt and Gordon [1985 (38)]. It had 16 sessions in a group format and was conducted by a psychologist and a psychiatrist from the therapeutic program. Thirdly, there was a social skills program based on the one developed in the MATCH project (39), which was also applied in a 12-week closed group format and conducted by a psychiatrist and a psychologist from the program. The fourth section was a consolidation of behavioral and lifestyle change programs. Nursing professionals developed this type of intervention in semi-closed groups (every 3 months, there were new additions), lasting 6 months. The last step was the preparation for discharge. The purpose of this program was to prepare patients for the cessation of treatment and to coordinate their referral to the Primary Care team. It lasted 6 months.

During the treatment period, patients attended weekly interviews with the nurse and were asked about their consumption during that week and whether or not they had participated in the mutual help group meetings. At each visit, the alcohol content in exhaled air was determined with a breathalyzer. During the two years of post-discharge follow-up, nurses telephoned the patient every two weeks to determine the number of consumption and days of attendance to the mutual aid groups.

Complete assessments occurred at two points: a) Baseline evaluation (T0), performed when the patients had completed detoxification and abstained for at least 3 months. In the previous 2 weeks, they were free from taking psychotropics (naltrexone, disulfiram, BDZ, anticonvulsants). Tests were performed in 2–3 days, and b) Two years after discharge (T2). At both times, the subjects completed an interview to collect clinical information, completed the psychological tests, had a blood test to determine blood values such as gamma-glutamyl transferase (GGT), and performed the psychophysiological evaluation (startle reflex modulation while visualizing ARS and salivary cortisol test).

Only abstinent patients were included at discharge. Researchers responsible for the psychological and neurobiological assessments were not blinded to the group to which the patient belonged or to the phase of the patient’s treatment.

### 2.3 Instruments of clinical evaluation

Patients were interviewed using the Structured Clinical Interview for DSM-5 (40) and diagnosed according to the criteria of the Diagnostic and Statistical Manual of Mental Disorders [DSM-5; APA 2013 (5)]. The assessment scales are specified below.

The Severity of Alcohol Dependence Scale, [SADS (41)] consists of 30 questions with four alternative answers. The total score differentiates between mild (score <20), moderate (21–37) and severe (>37) dependence.

Alcohol Timeline Followback (TLFB) was designed by Sobell and Sobell (42) to determine daily alcohol consumption retrospectively. This interview was used to determine the presence of relapses, the extent of alcohol consumption, and the periods of accumulated abstinence. Before the beginning of each group session, the treatment program nurse asked about consumption or abstinence days during the past week.

The Test for Detection of Cognitive Impairment in Alcoholism (TEDCA) is a brief, easy-to-apply and valuable tool for the detection of cognitive impairment (both moderate and mild) in a population with a history of alcohol use disorder. The TEDCA integrates the dimensions mainly affected by this type of patient: Visuospatial Cognition, Memory/Learning, and Executive Function. Each dimension has a maximum score of 6 points, the maximum test score being 18 points. The cut-off point for detecting a possible cognitive impairment is placed at 10.5 points, so those subjects who obtained a lower score were excluded from the study (15).

The Barrat Impulsivity Scale [BIS-11 (43)] is a 30-item self-report that determines impulsivity’s cognitive, motor, and lack of planning components. The Spanish version (44) has a good alpha coefficient and maintains the three initial factors.

The Hamilton Anxiety Rating Scale [HARS, Hamilton 1959 (45)] and the Hamilton Depression Rating Scale [HDRS, Hamilton 1967 (46)] were used to determine anxiety and depression symptoms, respectively.

The Self-Assessment Manikin (SAM) is a system used to assess the emotional response to a photograph using drawings provided by the International Affective Picture System (IAPS). The drawings are evaluated according to valence, arousal, and dominance. The valence part assesses the subject’s emotions from pleasant to unpleasant on a 1 to 9-point scale. The points increase from right to left (47).

In the different assessments, the baseline gamma-glutamyl-transferase (GGT) level was used as an indirect marker of alcohol consumption.

### 2.4 Psychophysiological evaluation/Cue reactivity

The startle reflex procedure was similar to the one used in previous studies (11). Participants were seated in comfortable chairs in front of high-resolution monitors, electrodes were attached, and signal quality was checked. Then, they were exposed to a set of 60-color photographs. The thirty alcohol-related images depicting alcoholic beverages or typical drinking situations were used in other similar studies (14, 48, 49). Pictures were randomly presented to each participant. Each image was displayed on the screen for six seconds, with a randomized inter-stimulus interval between 20–31 seconds and a blank screen after picture offset. Startle probes were presented 2.5 to 5 seconds after the onset of the visual stimulus (target startle) or 4 to 10 seconds after the beginning of the inter-stimulus interval. Each alcohol-related picture was exhibited 4 times during the experiment, and startle probes were presented in 50% of them.

Affective pictures were selected from the IAPS (50) according to their valence and arousal normative values (51, 52). The slides of pictures included 30 neutral pictures and 30 alcohol-related pictures. Neutral pictures (e.g., household and inanimate objects) had a mean valence of 5.01 [1.18] and a mean arousal of 2.10 [1.12]. We used different pictures for men and women but with similar valence and arousal scores. Alcohol-related pictures depicted alcoholic beverages (12 for beer, 10 for liquors) or typical drinking situations (6 in a bar, 2 with family members) as used in other studies (11, 15, 48). These images were standardized by Grusser et al., who developed a set of visual and olfactory stimuli specific for each substance (alcohol, tobacco, opiate and cannabis) for addicted patients and healthy individuals (53).

Three examples of the two slide categories were randomly allocated to 10 experimental blocks. The pictures were presented randomly within each block, composed of two different picture orders, and counterbalanced between participants within groups. Startle probes were presented 2.5 to 5 seconds after the onset of the visual stimulus (target startle) or 4 to 10 seconds after the beginning of the inter-stimulus interval. Each block had six intrastimulus (50%) and five interstimulus (50%) startles. All alcohol-related pictures were exhibited twice during the experiment, and startle probes were presented in 50% of them.

Startle data acquisition. The acoustic startle stimulus consisted of a 100 ms burst of white noise at 95dB, with instantaneous rise time [see ref (10, 11)]. The noise burst was presented binaurally with Sennheiser HD500 headphones (Wedemark, Germany). Each session began with 4 test startle-probe trials before the first picture was displayed. In addition, eight startle probes were presented during inter-picture intervals to enhance the unpredictability of the startle presentation. Electrodes (Ag–AgCl) filled with saline gel were attached to each participant’s right orbicularis oculi region to record startle eye blink electromyogram (EMG). This signal was acquired and amplified using an EMG100A module (BIOPAC Systems, Goleta, CA, United States) connected to an MP100WSW bioamplifier (BIOPAC Systems) with a constant time of 1000 ms. The EMG signal was recorded at a sampling rate of 1000 Hz with a low-pass filter of 500 Hz.

Startle responses were rejected under the following circumstances: a) the onset of the startle reflex did not occur within a period of 21 to 120 ms after the stimulus, b) EMG activity within the 20 ms baseline period was 12 μV or more (excessive noise), or c) the change from baseline to peak activity during the 21 to 120 ms after probe onset was less than 2 μV. Subjects were excluded if they had 25% or more invalid tests. Based on these exclusion criteria, startle data from 44 patients and 15 controls were not used.

The startle reflex response was measured in microvolts (μV).

Startle-probe outcomes: For this study, the EMG values obtained while viewing neutral and alcohol images were used. To compare the startle response after viewing alcohol images and the response after viewing neutral images, we did not use raw values but rather the difference between the baseline value (50 ms before the startle test) and the most significant amplitude of the startle between 5 and 150 ms after viewing the image (35, 54).

In this way, the amplitude measurements for each type of picture (neutral and alcohol) could be considered on the same scale.

After recording ARS, each participant was assessed for arousal, valence, and dominance, as evaluated with the Self-Assessment Manikin [SAM; Bradley and Lang (47)].

### 2.5 Cortisol reactivity after visualization of alcohol-related cues

Although some studies have used visualization of ARS alcohol-related stimuli as a stress test (34), and some studies have found that ARS elicits stress responses similar to those elicited by psychological stress tests (18), we have considered exposure to ARS as an appropriate approach of cortisol reactivity (19).

#### 2.5.1 Determination of cortisol pre (basal) and post-visualization of alcohol-related cues

Saliva was collected by the subjects using standard Eppendorf tubes (1.5ml, Eppendorf, Hamburg, Germany), stored at room temperature until the completion of the session and then kept at −20°C until analyses. After a biochemical analysis, the fraction of free cortisol in saliva (salivary cortisol) was determined using an RIA with salivettes (Sarstedt Inc. Texas). Inter- and intra-assay coefficients of variance were below 9%. All experimental sessions began at 13:00 and were completed by 15:30 to optimize the slow descent in cortisol levels at this time of day so that changes in salivary cortisol following the startle task (exhibition of alcohol-related cues) could be evaluated.

Upon arrival at the psychophysiology lab room, participants were asked to refrain from drinking beverages, eating, smoking, exercising and brushing their teeth for the 2-hour protocol. Water was available for all participants to drink.

### 2.6 Variables

#### 2.6.1 Biological markers of addiction

a) Cue-reactivity towards alcohol. Startle probe outcome after viewing alcohol-related images. b) Cortisol reactivity: Pre- and post-startle test salivary cortisol levels during which they view alcohol-related images. To determine the bluntness of the cortisol response to ARS alcohol-related stimuli, the difference between the post and pre-response to ARS alcohol-related stimuli was used. Thus, higher values indicated greater axis reactivity to the stimuli, while lower values indicated greater blunting.

#### 2.6.2 Psychological state

Scores on scales of anxiety, depression and impulsivity and the Valence, Arousal and Dominance of alcohol images assessed by the Self-Assessment Manikin (SAM).

#### 2.6.3 Indirect markers of alcohol consumption

GGT levels.

Days of consumption during the treatment period. Total number of days of abstinence during the first and second year of treatment.

### 2.7 Procedure

The research protocol for this study was approved by the Research Ethics Board of the 12 de Octubre University Hospital, and all participants signed an informed consent form on the day of their participation (Ceic: N° CEIm: 19/086). Once the criteria were met and participants signed the informed consent form, the characteristics of the psychological and psychophysiological evaluations were explained. Participants underwent the first assessment session after 8–12 weeks (mean: 28 days, range: 25–30 days) of giving up alcohol with supervised abstinence (information supplied by family members) (pre-test).

The study protocol was performed at baseline (T0) and two years after discharge (T2) and was carried out on 2–3 consecutive days (Information has been described in previous paragraphs – Experimental design).

### 2.8 Statistical analyses

The characteristics of the sociodemographic variables of the subjects according to the two population groups (cases and controls) will be summarized using the number of patients (n), the mean and standard deviation or absolute and relative frequency. A comparison of variables between populations was performed using Student’s t-test for independent samples and Pearson’s Chi-square test or Fisher’s Exact test.

Changes in the study variables in the patient group population (cases) at the two evaluation points, baseline and two years, were estimated using the Student’s t-test for related samples. In addition, a repeated measures model (MMRM) of variance was fitted to the log values over time. The model included the time point (time of measurement) as a fixed effect, adjusted for age, sex and days of alcohol consumption. An unstructured covariance matrix was used, and individual heterogeneity was incorporated. The results of this model were presented as the estimated mean value at two years, together with the 95% confidence interval.

All analyses were controlled for sex and days of alcohol consumption throughout the follow-up. As the control group was selected and matched based on variables such as age, sex, weight (body mass index) and number of cigarettes consumed, these variables were not controlled in comparison analyses between patients and controls.

All analyses were conducted using Stata Statistical Software Release 15 (StataCorp. 2017. Stata Statistical Software: Release 15. College Station, TX: StataCorp LLC) and a significance level of 5%.

## 3 Results

### 3.1 Sociodemographic differences between patients and controls

**Table 1** includes the sociodemographic variables of both groups. The patients were predominantly male, with an average age of 40 years, and 20% were unemployed. The patients who requested treatment for their alcohol dependence had a severe addiction (SADS>25). They started drinking alcohol at the age of 17 and met the criteria for alcohol dependence by the age of 30. They had an average of 4 previous relapses and a pre-study average alcohol consumption of 25 Standard Drinking Units (SDU, in Spain, the standard drink is equivalent to 10 grams of ethanol (30).

**Table 1:** Clinical sociodemographic differences between patients and control group.

Variable	Patient group (n=154)	Control group (n=138)	Signification
Age, x(DT) Gender (male), n(%)	42.18 (8.65) 99 (64.29)	40.94 (7.85) 88 (63.77)	T=1.203, df=290, p=0.230 X^2^ = 0.92, df=1, p=0.51
Educational level Primary Middle University	23 (14.93) 79 (51.29) 52 (33.76)	20 (14.49) 112 (81.16) 6 (4.35)	X^2^ = 41.64, df=2, p=0.00
Professional activity Unemployment, n(%)	30 (19.48)	23 (16.67)	X^2^ = 0.729, df=4, p=0.948
Age of onset of drinking, x(DT) Years of alcohol dependence, x(DT)	17.42 (5.33) 29.81 (14.59)	16.16 (2.29) –	T=2.589, df=290, p=0.010
Family history of alcoholism, n(%)	55 (35.71)	5 (6.75)	X^2^ = 45.91, df=1,p=0.001
Number of previous treatments for alcohol dependence, x(DT)	3.2 (0.21)	–	–
Previous relapses, x(DT) Alcohol and tobacco use Drinking units per drinking day, x(DT)	4.24 (1.76) 24.75 (21.89)	– 9.52 (0.31)	– – T=12.769, df=290, p= 0.00
Cigarettes/Day	21.46 (9.65)	20.14 (7.01)	T=0.817, df=290, p=0.414
Body Mass Index	23.18 (2.26)	23.16(2.30)	T=0.114, df=290, p=0.90
Psychiatric history Depressive disorders, n(%) Anxiety disorders, n(%) Personality disorders, n(%)	25 (16.23) 29 (18.83) 19 (12.33)	12 (8.69) 14 (10.14) 7 (5.07)	X^2^ = 3.73, df=1, p=0.053 X^2^ = 4.37, df=1, p=0.036 X^2^ = 4.33, df=1, p=0.029
Baseline assessments The Severity of Alcohol Dependence Scale, (SADS), x(DT)	28.80 (14.59)	–	–

Despite having excluded severe psychiatric comorbidities, anxiety disorders followed by depressive and personality disorders were present in 10–20% of the patients. Except for educational level, which was slightly higher in the patient group, there were no other significant sociodemographic differences between patients and controls.

Changes in psychophysiological and psychological variables at baseline (T0) and after two years (T2) of treatment. **Tables 2**–**4** show the changes after two years of treatment (T0 vs. T2) in the psychophysiological and psychological variables included in the study among the patient group (cases).

**Table 2:** Differences in patients’ biological and psychobiological variables throughout treatment (T0 vs. T2).

Univariate analysis			Analysis adjusted		
Parameters	Coefficient (CI 95%)	P value	Variable	Coefficient (CI 95%)	P value
Startle Alcohol	1.24 (-0.19;2.68)	0.091	two-year change	1.24 (-0.19;2.68)	0.091
			Sex (Men)	-0.16 (-2.19;1.88)	0.881
			Age	0.01 (-0.10;0.12)	0.843
			Days of alcohol consumption	-0.08 (-0.17;-0.00)	0.048
Startle Neutra	0.92 (-0.12;4.22)	0.43	two-year change	0.92 (-0.12; 4.22)	0.43
			Sex (Men)	-0.07 (-1.12;1.17)	0.78
			Age	-0.39 (-0.41;-0.01)	0.56
			Days of alcohol consumption	-0.01 (-0.08;0.05)	0.657
Cortisol Basal	-0.16 (-0.37;0.04)	0.115	two-year change	-0.17 (-0.37;0.04)	0.115
			Sex (Men)	-0.23 (-0.47;0.01)	0.060
			Age	0.01 (-0.01;0.02)	0.194
			Days of alcohol consumption	0.01 (-0.01;0.02)	0.107
Cortisol post task	0.13 (-0.04;0.31)	0.152	two-year change	0.13 (-0.04;0.31)	0.152
			Sex (Men)	-0.29 (-0.54;-0.04)	0.022
			Age	0.01 (-0.01;0.02)	0.195
			Days of alcohol consumption	-0.01 (-0.2;-0.00)	0.036

**Table 3:** Changes in psychological variables after two years of treatment in alcohol dependent patients (T0 vs T2).

Unvariate analysis			Analysis adjusted		
Parameters	Coeficient (CI 95%)	P value	Variable	Coefficient (CI 95%)	P value
Implusivity	-4.38 (-5.34;-3.41)	<0.001	Two-year change	-4.38 (-5.34;-3.41)	<0.001
			Sex (Men)	-1.04 (-4.61;2.52)	0.567
			Age	-0.01 (-0.21;0.19)	0.915
			Days of alcohol consumption	-0.11 (-0.26;-0.03)	0.123
Anxiety	-1.06 (-1.67;-0.48)	0.001	Two-year change	-1.06 (-1.67;-0.46)	0.001
			Sex (Men)	1.03 (-0.48;2.54)	0.180
			Age	-0.02 (-0.10;0.07)	0.691
			Days of alcohol consumption	-0.05 (-0.12;0.01)	0.078
Depression	-1.34 (-2.24;-0.45)	0.003	Two-year change	-1.34 (-2.24;-0.45)	0.003
			Sex (Men)	-0.28 (-1.79;1.25)	0.722
			Age	-0.04 (-0.12;0.05)	0.372
			Days of alcohol consumption	-0.06 (-0.12;0.01)	0.079
GGT	-4.82 (-8.19;-1.44)	0.005	Two-year change	-4.82 (-8.19;-1.44)	0.005
			Sex (Men)	-1.22 (-5.87;3,43)	0.607
			Age	0.08 (-0.17;0.34)	0.514
			Days of alcohol consumption	0.16 (-0.03;0.35)	0.130
Pleasantness	-1.04 (-1.33;-0.74)	<0.001	Two-year change	-1.04 (-1.33;-0.74)	<0.001
			Sex (Men)	-0.17 (-0.58;0,23)	0.401
			Age	-0.01 (-0.03;0.01)	0.465
			Days of alcohol consumption	-0.01 (-0.2;-0.02)	0.877
Arousal	-0.36 (-0.72;-0.01)	0.049	Two-year change	-0.36 (-0.72;-0.01)	0.049
			Sex (Men)	-0.34 (-0.73;0.04)	0.082
			Age	0.01 (-0.01;0.03)	0.455
			Days of alcohol consumption	0.01 (-0.03;-0.02)	0.781

**Table 4:** Differences in T2 in evaluation tests of patients and control group.

Variable	Patient group (n=154)	Control group (n=138)	Signification
Affective modulation of the startle reflex			
Startle viewing images of alcohol	47.99 (8.70)	54.86 (9.54)	T=6.43, df=290, p=0.00
Startle basal-neutras	62.88 (9.44)	60.21 (6.80)	T=2.74, df=290, p=0.00
Salivary cortisol			
Basal cortisol	6.61 (0.96)	5.61 (0.78)	T=9.56, df=290, p=0.00
Post-task cortisol	7.49 (0.94)	6.75 (0.80)	T=7.13, df=290, p=0.00
Proxy marker of alcohol consumption			
GGT levels, x(DT)	48.69 (16.43)	25.32 (8.49)	T=15.007, df=290, p=0.00
Psychological Assessments			
Barrat Impulsivity Scale, x(DT) Hamilton Anxiety Scale, x(DT) Hamilton Depression Scale, x(DT)	47.55 (10.12) 10.58 (3.59) 8.31 (3.50)	38.05 (11.92) 8.51 (1.91) 5.88 (1.98)	T=7.36, df=290, p=0.00 T=6.02, df=290, p=0.00 T=7.150, df=290, p=0.00
Scores on the Manikin scale in relation to alcohol imagery			
Pleasantness Arousal Dominance	5.35 (1.28) 2.78 (1.38) 2.39 (1.38)	3.22 (9.65) 2.60 (1.25) 1.71 (0.73)	T=15.11, df=290, p=0.00 T=1.16, df=290, p=0.00 T=5.16, df=290, p=0.00

### 3.2 Psychophysiological tests

#### 3.2.1 Modulation of the startle reflex after viewing ARS

No significant changes were observed in the magnitude of the startle response after viewing alcohol-related images in the T0 and T2 sessions (p=0.09). These results did not show any interaction between sex or age. Still, they did show an interaction with the number of days of alcohol consumption throughout the study (p=0.048), such that those who had consumed for more days exhibited a lower startle response when viewing alcohol-related images, which indicated that the appetitive salience towards alcohol-related stimuli was higher in these patients.

The response to neutral images did not change over time (p=0.43). The scores for men and women were similar. Males and females scored similarly. In this variable, there was no interaction with the number of days of alcohol consumption during the treatment period.

#### 3.2.2 Cortisol test before and after viewing ARS

The pre-cortisol measurements did not change significantly between T0 and T2 (p=0.115). There were no significant interactions with age, sex or the number of days of consumption during the two years. The cortisol measurements did not show significant differences after viewing alcohol-related images at T0 and T2 (p=0.152). However, in males (p=0.022) and in those who had consumed alcohol throughout the treatment (p=0.036), post-startle salivary cortisol levels were significantly lower. This lower reactivity after viewing alcohol-related cues indicated a more significant allostatic load.

A model was created to explain the blunting of cortisol reactivity (post-cortisol minus pre-cortisol), and sex and days of alcohol consumption were introduced as modulating variables. It became clear (F (2,151)=4.58, p=0.011) that in men, the difference in cortisol values was reduced by 0.37 points (p=0.048) and that for each day of alcohol consumption, the difference was reduced by 0.02 points (p=0.03).

#### 3.2.3 Scores on psychological scales

After two years of treatment, patients experienced a significant decrease in scores on anxiety, depression and impulsivity scales (see **Table 3**). These changes, which occurred in all three dimensions, were not influenced by sex, age or the number of days of alcohol consumption during treatment.

Concerning the subjective response of the valence, arousal and dominance of the alcohol-related slides, there was a significant decrease in these dimensions (p=0.001, p=0.045, p=0.027) with no significant interaction with sex, age or number of days of consumption.

#### 3.2.4 Differences in psychophysiological and psychological variables between patients at the end of the study (T2) and a control group

When the T2 evaluations of the patients were compared with those of a control group matched for age, sex, cigarette consumption and BMI, we observed (see **Table 4**) that the startle response was significantly lower in patients than in controls, indicating greater appetence towards ARS. Pre- and post-task cortisol levels were also significantly higher in patients than in controls.

The patients had statistically significantly higher scores on anxiety, depression and impulsivity scales. The gamma-glutamyl transpeptidase (GGT) values were also considerably higher in the patients than in the controls. That is to say, the situation at the end of the treatment (T2) indicated that the neurobiological (startle response and cortisol reactivity) and clinical (scales of impulsivity, negative emotionality, and subjective appetitiveness towards alcohol-related images) markers of addiction continued to differentiate the group of patients from the controls.

## 4 Discussion

To our knowledge, this is the first study to evaluate the long-term modifications (2 years) of two neurobiological markers triggered by the visualization of ARS together with psychological variables of negative emotionality in subjects with severe alcohol dependence after completing cognitive behavioral treatment.

The most relevant results were that: a) the startle reflex response after viewing ARS did not change significantly from the baseline (T0) to the final (T2) evaluation, but the days of alcohol consumption during the study decreased the magnitude of the response; b) pre- and post-task salivary cortisol values taken at T0 and T2 were similar. Men and patients who had consumed alcohol throughout the treatment had a lower cortisol reactivity (higher blunders) to the ARS; c) scores on the anxiety, depression and impulsivity scales showed a significant decrease between T0 and T2; d) the subjective evaluation of valence, arousal and dominance of alcohol-related slides decreased significantly between T0 and T2; e) after two years of treatment (T2), patients, compared to controls, still had significantly altered responses in all the neurobiological and negative emotionality variables evaluated during the study (motivational startle response, cortisol reactivity and negative emotionality). Considering that this is the first study to address the long-term changes in these variables, our hypotheses regarding the magnitude of the startle response and negative emotionality have been confirmed. Unexpectedly, the response to cortisol reactivity remained unmodified two years after treatment.

Regarding the modulation of the startle reflex after viewing ARS, our results support the idea that severe AUD is a chronic disorder with a tendency to relapse [DSM-5; APA 2013 (5)], basically because the motivational response remained unmodified after two years of cognitive-behavioral treatment. It has been shown that ARS may evoke a positive affective condition or facilitate focusing attention on reward-related stimuli, which can lead to a renewed search for the drug (8, 55). The fact that patients who had more days of alcohol consumption during the study presented a lower magnitude of the startle response, while the visualization of ARS supports the clinical experience that occasional consumption or relapses, produced during the treatment of patients with severe addiction, worsens the prognosis in the short and medium term, possibly due to the increase in the motivational value of the ARS caused by the consumption and relapses themselves (53, 56–58).

This persistent response could also be a precondition for these patients. Similar alterations have been found in subjects with a family history of alcoholism (59). However, no significant differences were found when we compared the results of the FHA+ group (n=55) with those of the other group (FHA-, n=99). Therefore, the most plausible hypothesis is that this persistent alteration of startle reflex modulation represents a state marker of patients with moderate-severe AUD (10, 11, 35, 53).

Considering that this objective assessment has been associated with craving self-reports, our findings contrast with those from studies that have shown a decrease in alcohol-craving scores during the treatment of alcohol-dependent patients [see review by Ray et al. (60)]. The results of this study support the hypothesis that there may be a dissociation between explicit and implicit stimulus processing (61). Our data are in line with an incentive-motivational model of drug cues (62, 63) and suggest that sensitization is the neurobiological correlate of an ‘addiction memory’ and may represent a process highly resistant to overwriting (53, 63–65).

Regarding cortisol reactivity, our results indicate that the pre-and post-task cortisol levels of the patients did not substantially change over time. Given that lower post-stress cortisol values may indicate a greater allostatic load on the stress axis, our results can be interpreted as indicating that greater exposure to alcohol cues (typical of males) and frequency of alcohol use contribute to the maintenance of this allostatic load (21, 25). Our results support the chronicity of severe cases of AUD (20, 21). However, no study has included such an extended follow-up of this variable. The study by Munro et al. (22), which included 18 male patients with periods of abstinence ranging from 7 days to 3.5 years of abstinence and a control group of 23 social drinkers, found the cortisol level in the patient group was higher compared to those of the social drinkers group, independently of years of abstinence.

The relationship between alcohol and cortisol has facilitated the development of a model that explains the role of glucocorticoids in alcohol consumption behavior based on changes in peripheral cortisol levels and responses across the entire spectrum of alcohol consumption levels (20). It has been noted that people with alcohol addiction or binge drinking have higher cortisol levels than those who drink moderately, indicating a change in the functioning of the HHA axis. This also suggests possible changes in the cerebral glucocorticoid pathways in humans, which may increase the risk of excessive alcohol consumption. It is known that alcohol consumption stimulates the release of cortisol (21); however, in response to stress or exposure to alcohol, the increase in cortisol is lower in subjects with AUD than in social drinkers. Thus, after drinking alcohol, alcohol-dependent drinkers do not feel its effects with the same intensity as those who drink moderately (21, 66). Given that cortisol is fundamental for survival, the responses to stress signals must be very well conserved in the cerebral stress pathways (2) in such a way that they can boost cortisol levels in response to stressful stimuli. In people who, like many of our patients, have blunted cortisol responses, this mechanism may indicate a greater motivation for alcohol consumption and thus increase cortisol levels (66). In other words, this blunting constitutes a neurophysiological impulse of motivation for alcohol to increase cortisol and the functioning of the HHA axis.

Alterations in the cortisol response may predate the development of alcohol use disorder. Since a history of a family history of alcoholism is associated with early adverse experiences and an increased stress response (67), we compared the group of patients with and without a family history of alcoholism and found no significant differences.

The results on the determinations of *negative emotionality* are in line with those of other long-term studies that indicated that anxiety, depression and impulsivity decreased in patients during the treatment period. However, without reaching the levels of the control group [see works by our group (30, 31, 68)]. The persistence of high negative emotionality after two years of treatment also helps to explain the chronicity of this disorder, although patients are abstinent for an extended period.

The lack of normalization of these scores in the patient group can be interpreted in two different, non-exclusive ways. High scores on anxiety, depression or impulsivity are a risk factor for the development of substance use disorders during adolescence, and therefore, the patients included in our study may have had elevated levels of these dimensions before the development of addiction (69, 70). However, on the contrary, this could also be explained because of the neurotoxic effects of alcohol (71, 72).

Although we did not use neuroimaging techniques in our study, other studies have confirmed that the processing of negative emotions is altered in withdrawn alcohol-dependent patients [see review in Voon et al. (8)]. The anticipation of an adverse event, as an unpredictable threat, causes a reduced activation in the striatal corticolimbic regions of abstinent men with AUD, and negative contexts further decrease this neuronal activity during behavioral control tasks. In addition, negative emotional states can increase craving and alcohol consumption due to the hypoactivity of the striatal-corticolimbic connections and the difficulty in implementing behavioral inhibition strategies (8).

### 4.1 Clinical implications

We believe these results have several important implications for treating these patients. Firstly, he model of addiction as a brain disease best explains our results (65, 73, 74). In this model, the reward circuit is “hacked by alcohol” in such a way that alcohol and the signals associated with it acquire chronic survival value. This implies that the coping strategies to achieve and maintain abstinence must be prolonged and adapted to a sober lifestyle (75, 76). Given that, after having achieved sustained remission, patients continue to retain the vulnerability factors related to the response to ARS, in addition to the use of pharmacological and psychotherapeutic strategies that facilitate the detection of signs of negative emotionality and the implementation of behavioral inhibition skills (12, 77, 78). Different pharmacological alternatives to reduce the high stress of these patients should also be addressed (79).

Given that, a high stress response has been associated with frequent medical and psychological problems, and given that determining salivary cortisol levels is a simple test, it has been proposed to use this type of test to monitor the onset of stress-related diseases (endocrine and oncological diseases) (80).

This study has several limitations. These are patients with severe AUD who seek treatment, so our results do not apply to patients with mild-moderate dependence or to those who do not seek treatment. Most of the patients had family support, so we do not know if the course of these variables would have been the same in other conditions of lack of support.

Self-reports were used to evaluate consumption during the treatment and follow-up, complemented by alcohol determination in exhaled air and GGT determinations in blood samples. Although this methodology is widely contrasted (81), more objective techniques such as EtG (in urine or hair) could help corroborate self-reports (82).

Given the significant number of patients who could not undergo these tests, it can be hypothesized that the changes observed in this study correspond to the group with the least medical and psychiatric comorbidity and, therefore, *a priori*, those with the best prognosis.

The alterations found in the startle response and cortisol reactivity may be present in AUD subjects before the development of addiction, so follow-up studies of these markers should be conducted in early-stage subjects to test these assumptions. The cortisol response of subjects with alcohol use disorders may be altered before the development of the alcohol use disorder.

The strengths of this study are the large number of patients included, the use of a wide range of assessment tools, which has allowed us to evaluate different areas of addiction such as emotional reactivity, stress response and negative emotionality, the long follow-up period considered, and the comparison with a control group.

## Data Availability

The raw data supporting the conclusions of this article will be made available by the authors, without undue reservation.
